# Early career psychiatrists’ attitudes towards electroconvulsive therapy: an international cross-sectional survey

**DOI:** 10.1192/j.eurpsy.2023.2163

**Published:** 2023-07-19

**Authors:** C. Tapoi, C. Noel, R. de Filippis, D. Gurrea Salas, K. Mieze, D. Almeida, A. Pushko, M. Pinto da Costa

**Affiliations:** 1 General Psychiatry, Alexandru Obregia Clinical Psychiatry Hospital, Bucharest, Romania; 2 Child Psychiatry, Centre Hospitalier Universitaire Saint-Pierre, Université Libre de Bruxelles, Brussels, Belgium; 3 Psychiatry Unit, Department of Health Sciences, University Magna Graecia of Catanzaro, Catanzaro, Italy; 4 Department of Addictive Disorders, Psychiatric Services Aargau, Brugg, Switzerland; 5 Department of Doctoral Studies, Riga Stradins University, Riga, Latvia; 6 Department of Psychiatry and Mental Health, Hospital de Loures, Loures, Portugal; 7 Department of Psychiatry, Narcology and Medical Psychology, Ivano-Frankivsk National Medical University, Ivano-Frankivsk, Ukraine; 8 Institute of Psychiatry, Psychology & Neuroscience, King’s College London, London, United Kingdom

## Abstract

**Introduction:**

With a history of several decades, electroconvulsive therapy (ECT) has been carefully investigated and data supports its use as a safe and effective treatment for patients with severe depression, prolonged or severe manic episodes and catatonia. However, ECT is still regarded with reluctance by patients and caregivers, and its acceptance and use seem to be controversial even for psychiatrists.

**Objectives:**

To investigate the access to opportunities of training in ECT among early career psychiatrists and their views regarding the place of ECT in modern psychiatry.

**Methods:**

A cross-sectional study was conducted between July and December 2022 utilizing an anonymous online survey consisting of 36 multiple-choice and Likert scale questions.

**Results:**

These preliminary findings show a great discrepancy regarding the availability of ECT training in European countries, as access to specialized ECT centers is unavailable in some areas. Early career psychiatrists who had access to ECT training are more knowledgeable about the indications, precautions and side effects of this method. Most of our respondents consider ECT both an effective and a safe treatment option and have expressed their wish to improve their theoretical and practical competencies in ECT.

**Conclusions:**

ECT is a standard treatment and a therapeutic mainstay in psychiatry but is being less performed in some countries. Early career psychiatrists lack experience with ECT but are interested in training opportunities. Future actions are needed for the improvement of education and training in ECT.

**Disclosure of Interest:**

None Declared

